# Cyst of the Pancreas

**Published:** 1882-05

**Authors:** 


### CYST OF THE PANCREAS.

Dr. Nathan Bozeman presented a cyst of the pancreas, which had been mistaken before removal for ovarian cyst.

The patient was the wife of a physician of Texas, 41 years old, robust, and weighed 200 pounds. She had been perfectly healthy up to seven years ago, when she began to suffer from pain in the right inguinal region, hip, and thigh, and her abdomen began to enlarge.

This gradually progressed up to six or seven months ago, when she was examined by an eminent physician of New Orleans, who diagnosed ovarian cyst. She was subsequently sent to me, and I also thought the tumor ovarian, and decided on operation. This was done December

2d. Instead of ovarian cyst I found the tumor attached to the pancreas at junction of outer one third and inner two thirds.

The patient had been prepared for the operation by administration of salicin 15 grs. t. i. d., and was thoroughly cinchonized the day of the operation.

The patient after operation progressed as satisfactorily as any case of ovariotomy. There had been no symptom of interference with the function of the pancreas before operation.—*Proceedings N. Y. Pathological Society, in Med. Gazette.*

